# Thyroid cancer among children and adolescents aged 5–19 years from 1990 to 2021: a global, regional, and national perspective

**DOI:** 10.3389/fonc.2026.1694476

**Published:** 2026-02-19

**Authors:** Feng Wang, Jie Zhang, Lihua Fan, Weijuan Jiang, Shuixiu Yu

**Affiliations:** 1 Department of Radiotherapy, Jingjiang People’s Hospital Affiliated to Yangzhou University, Jingjiang, Jiangsu, China; 2 Department of Oncology, Jingjiang People’s Hospital Affiliated to Yangzhou University, Jingjiang, Jiangsu, China

**Keywords:** children and adolescents, DALYs, epidemiology, health inequality, incidence, thyroid cancer, trend in global burden

## Abstract

**Introduction:**

Thyroid cancer in children and adolescents (caTC) is characterized by a higher degree of malignancy, as well as an increased risk of distant metastasis and recurrence compared to thyroid cancer in adults. At present, new analyses of the global burden of caTC are limited and outdated. To conduct a comprehensive and systematic investigation into the burden and trends of caTC across the global population.

**Methods:**

Data were gathered from the Global Burden of Disease (GBD) study in 2021. A detailed analysis was performed using age-standardized incidence rate (ASIR), age-standardized prevalence rate (ASPR), Age-Standardized Disability-Adjusted Life Years (ASDR), and average annual percent change (AAPC). Joinpoint regression, cross-country inequality, decomposition, and frontier analyses were conducted.

**Results:**

In 2021, we discovered a total of 4, 951.78 new caTC cases globally, along with 30, 499.94 DALYs. The age-standardized rates (ASRs) were 0.24 per 100, 000 population for incidence and 1.52 per 100, 000 for DALYs. In trend analysis, the global incidence (AAPC, 1.00) and prevalence (AAPC, 1.05) exhibited an increasing pattern, while the DALYs (AAPC, -0.37) declined. The ASIR of caTC in Africa, America, and Asia demonstrated a continuous upward trend, whereas Europe witnessed a decline. Consistently, the ASIR, ASPR, and ASDR of caTC were all higher among women compared to men. Notably, countries with low SDI bore a higher disease burden. Population growth and epidemiological changes were the primary drivers of this increased disease burden. Despite different levels of development, many countries still had considerable potential to reduce these disease burdens.

**Conclusions:**

CaTC persists as a significant public health concern globally. Its burden varies considerably in terms of global distribution and magnitude, thereby emphasizing the necessity for the formulation of targeted regional and population-based policies aimed at primary prevention.

## Introduction

Thyroid cancer represents a prevalent endocrine malignancy among children and adolescents, and its incidence has been on the rise over the past few decades (1–3). The 2015 guidelines from the American Thyroid Association (ATA) recommend defining individuals aged 18 years and younger as children and adolescents (4). According to data from the National Cancer Institute in the United States, the annual incidence rate of thyroid cancer among this age group is 0.54 cases per 100, 000 individuals (5). Notably, within the 15–19 age bracket, thyroid cancer ranks as the eighth most prevalent malignancy overall and holds the second highest position among females, with a concerning upward trend observed over the years (6–8).

The characteristics of thyroid cancer in children and adolescents differ markedly from those observed in adults, including molecular, pathological, and clinical presentations (9, 10). CaTC is distinguished by its multifocal nature and aggressiveness, frequently extending beyond the thyroid capsule to involve vital structures such as the recurrent laryngeal nerve, blood vessels, esophagus and trachea. Moreover, at diagnosis, caTC shows a greater inclination toward distant and lymph node metastases, with prevalence rates ranging from 40% to as high as 80%. Distant metastases predominantly affect the lungs, with the bones and brain following in frequency (11–13). Although the occurrence of caTC is markedly lower compared to adults, it is characterized by a higher degree of malignancy and an elevated risk of distant metastasis and recurrence, thus necessitating increased vigilance and attention.

The Global Burden of Disease (GBD) initiative has become widely recognized as a tool for assessing the impact of various diseases worldwide. The latest version, GBD 2021, encompasses data on 371 diseases and injuries, spanning 204 countries and territories over the period from 1990 to 2021 (14). As far as we are aware, no extensive GBD examination has been undertaken to assess the global burden of thyroid cancer in children and adolescents within the 5–19 year age bracket. Hence, our objective is to systematically evaluate the trends in thyroid cancer burden for this age group by integrating various indicators from the GBD study, such as Age-Standardized Incidence Rate (ASIR), Age-Standardized Prevalence Rate (ASPR), Age-Standardized Disability-Adjusted Life Years (DALYs) rate, Estimated Annual Percentage Change (EAPC), and Average Annual Percent Change (AAPC). This will provide novel perspectives on the scale and distribution of the global burden of caTC. Our findings will not only inform regions with a heavy caTC burden but also contribute to the development of existing disease control and prevention guidelines across various countries and regions.

## Materials and methods

### Data source

The data employed in this analysis were sourced from GBD 2021, which covers a wide range of geographical scales, from the global to the continental level (Africa, the Americas, Asia, Europe), and down to the national level, encompassing 204 countries and regions. In GBD 2021, thyroid cancer was coded as C73 in ICD-10 (14, 15). Thyroid cancer in children was typically defined as occurring in individuals aged 18 years and younger. Therefore, we sought data on thyroid cancer in children and adolescents aged 5–19 years from 1990 to 2021. In this study, we obtained estimates for the ASIR, ASPR, and DALYs of caTC, and calculated the 95% uncertainty intervals (UI) for these estimates.

The 2021 GBD study generates consistent estimates of worldwide disease burden by integrating multiple data sources such as cancer registries, vital statistics, hospital records, population surveys, and peer-reviewed publications. Data completeness and quality vary substantially across countries, with more pronounced limitations in resource-constrained settings. The GBD consortium addresses these data gaps through advanced statistical modeling and imputation. All estimates are reported with 95% uncertainty intervals (UIs) to account for errors from sampling, modeling, and data limitations. These de-identified aggregated datasets are publicly available through the Global Health Data Exchange (GHDx) and received an informed consent exemption from the University of Washington Institutional Review Board (16).

### Temporal trend analysis

We used the Joinpoint regression model (version 5.1.0.0, National Institute of Health, Bethesda, MD, United States) to analyze temporal trends of ASIR, ASPR, and ASDR for caTC at global, continental, and national levels (17). Joinpoint methodology identifies pivotal trends’ inflection points, dividing the trend into segments. Subsequently, the epidemiological trends within each segment can be evaluated by computing the Annual Percent Change (APC) with a 95% Confidence Interval (CI) (18). To assess trends over specific time intervals (1990-1999, 2000-2009, 2010-2021, 1990-2021), we computed the AAPC. The AAPC serves as a summary measure and and was derived by calculating the weighted average of APCs, taking into account the duration of each segment.

### Cross-country inequality analysis

The Slope Index of Inequality (SII) and the Concentration Index (CI) served as standardized measures for assessing absolute and relative gradient inequalities, respectively, and were utilized to evaluate the disparities in the incidence, prevalence, and DALYs among different countries for caTC (19). In clinical terms, these indices help quantify how fairly the caTC burden is distributed across the development spectrum. The SII represents the absolute difference in DALY rates between the most and least advantaged countries in terms of SDI. A negative SII value indicates that the disease burden is disproportionately concentrated in lower-SDI countries. The CI measures relative inequality; a negative CI signifies that the burden is more heavily borne by populations in lower-SDI countries (20). The SII was calculated using regression analysis linking DALYs to a country’s median SDI, with a weighted model to address heteroscedasticity. The CI was computed by numerically integrating the area beneath the Lorenz curve, which represents the cumulative proportion of DALYs relative to the cumulative distribution of the population arranged according to SDI (21). Detailed SDI values for each country were presented in **Supplementary Table S1**.

### Decomposition analysis and frontier analysis

From 1990 to 2021, we used decomposition analysis to identify the key factors driving changes in caTC. Our objective was to measure the individual effects of epidemiological change, aging and population growth. Herein, ‘epidemiological change’ refers to the change attributable to variations in age-specific rates, capturing the net change in disease risk after accounting for the demographic influences of population aging and growth. The methodology involved isolating the influence of each component by holding the other two constant during the assessment (22, 23). We employed frontier analysis to examine the association between caTC burden and sociodemographic development. Using non-parametric data envelopment analysis, we established a nonlinear performance frontier representing the lowest achievable ASRs at each SDI level. Countries reaching this frontier serve as efficiency benchmarks, having attained the minimal disease burden for their developmental status. The “unfulfilled health improvement potential” for a country is then calculated as the difference between its observed ASR and the frontier-predicted ASR at its specific SDI, representing the scope for further burden reduction (24, 25).

### Statistics

A P-value less than 0.05 was deemed statistically significant. All statistical computations and data visualizations were carried out utilizing the Health Inequality Assessment Toolkit of the WHO, the joinpoint regression program (version 5.1.0.0), and R software (version 4.3.3). The analyses leveraged several key R packages, including ggplot2 (v3.5.1) and dplyr (v1.1.4) for data management, statistical modeling, and figure generation.

## Results

### Incidence, prevalence, DALYs of TC in children and adolescents

**Figure 1:**
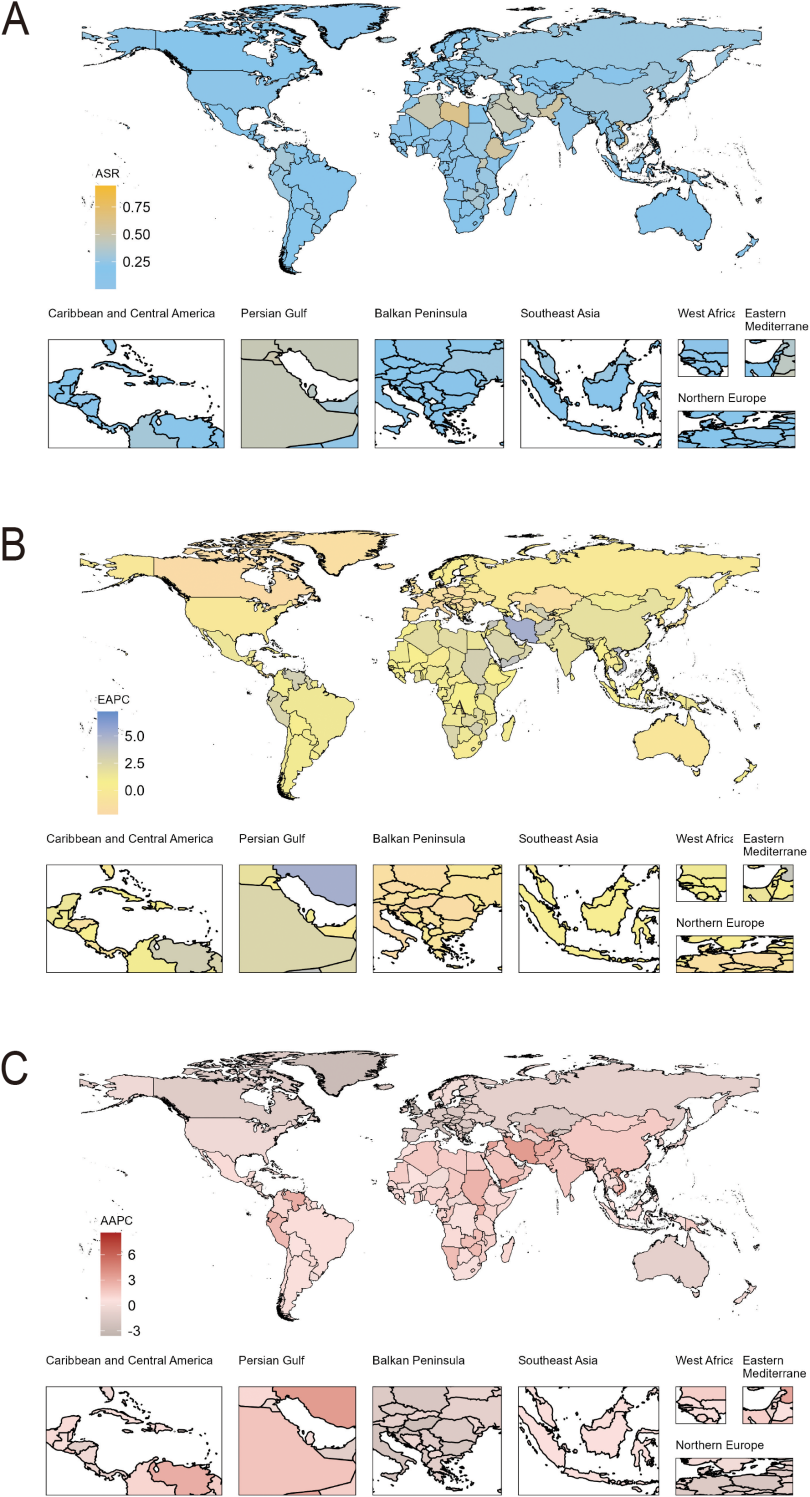
The incidence of caTC per 100, 000 population across 204 countries and territories using multiple indicators. **(A)** Age-standardised rate in 2021; **(B)** Estimated annual percentage changes (EAPCs) from 1990 to 2021; **(C)** Average annual percentage changes (AAPCs) from1990 to 2021.

According to the GBD 2021 data, there were 4, 905.97 new cases of caTC recorded globally. South Asia had the most new cases, whereas Oceania had the least. Globally, the incidence ASR per 100, 000 population was 0.24, showing significant variation across countries, ranging from 0.001 in Tajikistan to 0.94 in Niue. Geographically, the highest incidence was observed in North Africa and Middle East (ASR, 0.33), while the lowest was in Western Sub-Saharan Africa (ASR 0.04). When it came to gender disparities, the global average incidence rate for females (ASR, 0.34) was approximately 2.3 times that of males (ASR, 0.15), suggesting a potential sex-specific effect of caTC (**Figure 1**).

Globally, the total number of caTC cases was 44, 267.73, with South Asia topping the list with 14, 679.09 cases and Oceania having the least with 69.45 cases. Regarding prevalence, the global average ASR was 2.21 per 100, 000 people, with a wide range from 0.36 in Western Sub-Saharan Africa to 3.04 in North Africa and the Middle East. In terms of gender, the global prevalence rate for females (ASR, 3.11) was approximately twice that of males (ASR, 1.36). Considering the SDI, both the incidence and prevalence ASRs were significantly higher in regions with high-middle and middle SDI levels.

For DALYs, according to 2021 estimates, a total of 30499.94 years were lost globally. South Asia accounted for the highest number of DALYs, totaling 12, 823.15, while Australasia had the lowest, with just 20.14 DALYs. The global ASR of DALYs was 1.52 per 100, 000 population, with a significant variation ranging from 0.01 in Tajikistan to 6.10 in Tokelau. At the regional level, the rate of DALYs in Eastern Sub Saharan Africa was the highest (ASR, 3.54), while the lowest was in Australasia (ASR, 0.35). The DALYs ASRs for females (ASR, 1.87) were about 1.57 times those of males (ASR, 1.19). In terms of SDI, the ASR of DALYs was relatively higher in regions with a low SDI (**Table 1**, **Supplementary Table S2**).

**Table 1:** Incidence, prevalence, and DALYs of caTC in 2021, with ASR by sex and GBD region.

Regions	Incidence						Prevalence						Disability-adjusted life years (DALYs)					
	Both sexes		Female		Male		Both sexes		Female		Male		Both sexes		Female		Male	
	Cases (95% UI)	ASR per 100000 (95% UI)	Cases (95% UI)	ASR per 100000 (95% UI)	Cases (95% UI)	ASR per 100000 (95% UI)	Cases (95% UI)	ASR per 100000 (95% UI)	Cases (95% UI)	ASR per 100000 (95% UI)	Cases (95% UI)	ASR per 100000 (95% UI)	Cases (95% UI)	ASR per 100000 (95% UI)	Cases (95% UI)	ASR per 100000 (95% UI)	Cases (95% UI)	ASR per 100000 (95% UI)
Global	4905.97 (3955.88-6280.15)	0.24 (0.20-0.31)	3352.13 (2585.24-4585.43)	0.34 (0.27-0.47)	1553.84 (1187.93-1880.61)	0.15 (0.12-0.18)	44267.73 (35718.26-56613.59)	2.21 (1.78-2.82)	30337.03 (23392.49-41478.16)	3.11 (2.40-4.25)	13930.69 (10653.23-16853.92)	1.36 (1.04-1.64)	30499.94 (23930.77-39717.25)	1.52 (1.19-1.98)	18278.01 (13402.93-26137.70)	1.87 (1.37-2.67)	12221.93 (9056.01-14941.04)	1.19 (0.88-1.46)
Socio-demographic index																		
High	426.28 (394.99-468.51)	0.23 (0.21-0.25)	225.59 (206.17-257.04)	0.25 (0.23-0.29)	200.69 (179.87-226.88)	0.21 (0.19-0.24)	3911.26 (3623.60-4298.30)	2.12 (1.96-2.33)	2069.85 (1891.66-2358.17)	2.30 (2.11-2.62)	1841.41 (1649.78-2081.34)	1.95 (1.74-2.20)	1035.88 (930.70-1179.23)	0.56 (0.51-0.64)	468.13 (408.69-556.60)	0.52 (0.46-0.62)	567.76 (504.76-660.34)	0.60 (0.53-0.70)
High-middle	643.41 (532.51-801.47)	0.27 (0.23-0.34)	364.98 (290.62-482.17)	0.33 (0.26-0.43)	278.43 (202.97-371.80)	0.23 (0.17-0.30)	5885.03 (4871.60-7333.57)	2.51 (2.08-3.13)	3342.32 (2661.88-4413.74)	3.00 (2.39-3.96)	2542.71 (1853.61-3397.58)	2.07 (1.51-2.77)	2017.99 (1637.26-2500.33)	0.86 (0.70-1.07)	907.37 (707.88-1234.47)	0.81 (0.63-1.11)	1110.63 (822.08-1437.95)	0.91 (0.67-1.17)
Middle	1560.29 (1207.20-1958.31)	0.27 (0.23-0.34)	975.18 (728.24-1304.43)	0.35 (0.26-0.47)	585.11 (400.47-767.78)	0.19 (0.13-0.26)	14177.91 (10961.48-17797.69)	2.44 (1.89-3.06)	8889.39 (6637.66-11893.30)	3.17 (2.37-4.24)	5288.52 (3613.43-6941.24)	1.76 (1.20-2.32)	7209.34 (5595.25-8947.06)	1.24 (0.96-1.54)	3500.16 (2624.62-4757.41)	1.25 (0.94-1.70)	3709.18 (2601.78-4717.70)	1.24 (0.87-1.58)
Low-middle	1372.15 (1028.87-1994.03)	0.23 (0.17-0.34)	1083.51 (773.58-1687.24)	0.38 (0.27-0.58)	288.64 (210.62-373.10)	0.10 (0.07-0.13)	12273.64 (9199.87-17877.95)	2.08 (1.56-3.03)	9741.28 (6967.42-15172.34)	3.37 (2.41-5.24)	2532.36 (1853.05-3269.90)	0.85 (0.62-1.10)	10820.54 (8111.08-15402.44)	1.84 (1.38-2.62)	7362.78 (5158.28-11446.73)	2.55 (1.79-3.96)	3457.76 (2498.63-4535.44)	1.16 (0.84-1.53)
Low	900.70 (658.38-1305.57)	0.22 (0.16-0.32)	700.78 (487.54-1085.97)	0.35 (0.24-0.54)	199.91 (138.55-264.71)	0.10 (0.07-0.13)	7991.58 (5853.37-11626.71)	1.95 (1.43-2.84)	6275.30 (4365.61-9728.22)	3.10 (2.15-4.80)	1716.28 (1190.87-2273.37)	0.82 (0.57-1.08)	9395.16 (6845.14-13078.98)	2.28 (1.66-3.18)	6028.55 (4194.89-9332.32)	2.97 (2.07-4.60)	3366.61 (2313.35-4527.79)	1.60 (1.10-2.15)
Regions																		
Andean Latin America	44.51 (32.44-62.51)	0.25 (0.18-0.35)	27.49 (17.98-41.76)	0.32 (0.21-0.49)	17.02 (11.16-25.41)	0.19 (0.12-0.28)	402.98 (293.41-566.09)	2.28 (1.66-3.20)	250.23 (163.48-380.24)	2.93 (1.92-4.46)	152.75 (100.29-228.18)	1.67 (1.09-2.50)	262.55 (198.60-347.54)	1.49 (1.12-1.97)	120.45 (83.31-172.09)	1.41 (0.98-2.02)	142.10 (99.75-199.61)	1.56 (1.09-2.19)
Australasia	8.17 (5.99-11.07)	0.14 (0.10-0.19)	2.97 (2.16-3.99)	0.11 (0.08-0.14)	5.20 (3.55-7.36)	0.18 (0.12-0.25)	74.71 (54.67-101.43)	1.30 (0.95-1.76)	27.24 (19.79-36.59)	0.97 (0.70-1.30)	47.47 (32.29-67.35)	1.61 (1.09-2.28)	20.14 (15.36-26.25)	0.35 (0.27-0.46)	5.97 (4.32-8.13)	0.21 (0.15-0.29)	14.17 (10.65-18.62)	0.48 (0.36-0.63)
Caribbean	20.72 (15.20-26.70)	0.18 (0.13-0.23)	14.65 (9.77-20.44)	0.25 (0.17-0.36)	6.07 (4.36-8.32)	0.10 (0.07-0.14)	185.58 (136.73-238.72)	1.60 (1.18-2.06)	131.97 (88.38-183.88)	2.29 (1.53-3.21)	53.62 (38.76-72.94)	0.92 (0.67-1.26)	176.48 (113.54-250.88)	1.54 (0.99-2.18)	107.34 (57.60-173.03)	1.88 (1.01-3.04)	69.14 (45.03-106.69)	1.20 (0.78-1.86)
Central Asia	24.93 (20.71-29.99)	0.10 (0.09-0.12)	16.55 (13.62-20.06)	0.14 (0.12-0.17)	8.39 (6.72-10.27)	0.07 (0.05-0.08)	226.23 (188.10-272.00)	0.94 (0.79-1.13)	150.75 (124.05-182.90)	1.31 (1.08-1.59)	75.49 (60.54-92.33)	0.59 (0.48-0.73)	159.26 (135.29-189.62)	0.66 (0.56-0.78)	75.78 (64.37-89.19)	0.66 (0.56-0.78)	83.48 (69.23-101.00)	0.65 (0.54-0.79)
Central Europe	28.05 (24.49-31.54)	0.16 (0.14-0.17)	19.27 (16.35-21.99)	0.22 (0.19-0.25)	8.78 (7.32-10.55)	0.09 (0.08-0.11)	256.61 (224.08-288.50)	1.42 (1.24-1.60)	176.63 (149.92-201.55)	2.02 (1.72-2.31)	79.99 (66.70-96.02)	0.86 (0.71-1.03)	94.28 (83.08-107.37)	0.52 (0.46-0.60)	46.87 (41.10-54.22)	0.54 (0.47-0.62)	47.41 (40.19-56.05)	0.51 (0.43-0.60)
Central Latin America	132.31 (114.27-152.77)	0.20 (0.17-0.23)	80.52 (69.12-94.03)	0.24 (0.21-0.28)	51.79 (43.20-61.80)	0.15 (0.13-0.18)	1196.67 (1032.42-1383.24)	1.79 (1.54-2.07)	731.19 (627.50-854.75)	2.21 (1.90-2.59)	465.48 (387.83-556.21)	1.37 (1.14-1.64)	762.89 (669.54-873.30)	1.14 (1.00-1.31)	343.14 (301.43-393.81)	1.04 (0.92-1.20)	419.75 (360.02-492.56)	1.24 (1.07-1.46)
Central Sub Saharan Africa	26.42 (17.25-42.07)	0.05 (0.03-0.08)	16.41 (9.04-30.32)	0.07 (0.04-0.12)	10.00 (6.04-16.14)	0.04 (0.02-0.06)	232.85 (151.03-372.91)	0.45 (0.29-0.73)	147.01 (80.72-271.84)	0.58 (0.32-1.08)	85.84 (51.70-139.59)	0.33 (0.20-0.53)	353.71 (237.39-537.55)	0.69 (0.46-1.05)	164.88 (93.52-300.54)	0.65 (0.37-1.20)	188.83 (115.76-297.60)	0.72 (0.44-1.13)
East Asia	777.89 (572.53-1029.71)	0.30 (0.22-0.39)	386.54 (283.13-604.05)	0.32 (0.23-0.49)	391.36 (224.55-570.53)	0.28 (0.16-0.41)	7102.83 (5231.98-9404.99)	2.70 (1.99-3.57)	3536.14 (2589.73-5528.97)	2.88 (2.11-4.51)	3566.69 (2046.38-5200.37)	2.54 (1.46-3.70)	2731.70 (1887.24-3672.09)	1.04 (0.72-1.39)	991.25 (708.82-1547.60)	0.81 (0.58-1.26)	1740.45 (973.68-2551.54)	1.24 (0.69-1.81)
Eastern Europe	94.91 (86.85-104.61)	0.27 (0.24-0.29)	64.33 (57.89-72.37)	0.37 (0.33-0.42)	30.58 (27.22-34.32)	0.17 (0.15-0.19)	868.22 (794.26-957.25)	2.42 (2.22-2.67)	589.89 (530.85-664.33)	3.38 (3.04-3.81)	278.33 (247.87-312.21)	1.52 (1.35-1.70)	275.66 (247.89-309.63)	0.77 (0.69-0.86)	136.24 (119.76-157.84)	0.78 (0.69-0.90)	139.42 (122.97-158.28)	0.76 (0.67-0.86)
Eastern Sub Saharan Africa	518.34 (360.62-825.96)	0.32 (0.22-0.51)	404.51 (261.42-698.09)	0.50 (0.33-0.87)	113.83 (69.61-163.15)	0.14 (0.09-0.20)	4595.55 (3195.26-7330.50)	2.85 (1.98-4.54)	3620.81 (2340.84-6234.21)	4.51 (2.91-7.76)	974.74 (595.97-1398.18)	1.19 (0.73-1.71)	5727.93 (3906.50-8571.70)	3.54 (2.41-5.30)	3728.44 (2447.94-6367.76)	4.64 (3.05-7.93)	1999.48 (1203.96-2840.60)	2.44 (1.47-3.47)
High-income Asia Pacific	65.90 (56.12-79.08)	0.26 (0.22-0.31)	37.06 (32.00-46.75)	0.30 (0.26-0.38)	28.84 (22.07-38.25)	0.22 (0.17-0.30)	604.98 (515.25-726.14)	2.40 (2.04-2.88)	340.21 (293.76-429.19)	2.76 (2.38-3.48)	264.77 (202.48-351.57)	2.06 (1.57-2.73)	157.16 (135.63-191.04)	0.63 (0.54-0.76)	74.48 (62.40-96.57)	0.61 (0.51-0.79)	82.69 (70.16-106.10)	0.64 (0.55-0.83)
High-income North America	161.72 (152.11-172.27)	0.22 (0.21-0.24)	82.16 (77.68-87.10)	0.23 (0.22-0.25)	79.56 (72.76-87.40)	0.22 (0.20-0.24)	1484.17 (1395.87-1580.98)	2.06 (1.93-2.19)	753.97 (712.82-799.26)	2.13 (2.01-2.26)	730.19 (667.62-802.07)	1.99 (1.82-2.19)	381.25 (349.11-421.71)	0.53 (0.49-0.59)	167.22 (151.11-186.76)	0.47 (0.43-0.53)	214.03 (194.52-237.87)	0.59 (0.53-0.65)
North Africa and Middle East	579.40 (465.94-726.64)	0.33 (0.27-0.42)	364.54 (278.10-493.48)	0.43 (0.33-0.59)	214.87 (149.75-283.75)	0.24 (0.17-0.31)	5303.88 (4265.37-6652.94)	3.04 (2.45-3.81)	3338.56 (2545.67-4520.10)	3.96 (3.02-5.36)	1965.32 (1369.10-2595.85)	2.18 (1.52-2.88)	1796.75 (1426.00-2241.48)	1.03 (0.82-1.28)	945.06 (722.63-1306.97)	1.12 (0.86-1.55)	851.69 (613.96-1124.55)	0.94 (0.68-1.25)
Oceania	7.85 (4.59-11.75)	0.18 (0.10-0.27)	4.22 (2.19-6.87)	0.20 (0.10-0.33)	3.62 (1.99-6.11)	0.16 (0.09-0.26)	69.45 (40.77-104.68)	1.58 (0.93-2.38)	38.13 (19.73-61.91)	1.83 (0.95-2.97)	31.32 (17.36-53.85)	1.35 (0.75-2.32)	81.89 (46.76-124.66)	1.86 (1.06-2.83)	25.76 (13.39-40.50)	1.23 (0.64-1.94)	56.13 (29.89-94.86)	2.42 (1.29-4.09)
South Asia	1641.73 (1173.69-2450.19)	0.29 (0.21-0.44)	1374.82 (946.06-2140.59)	0.51 (0.35-0.79)	266.91 (174.68-362.26)	0.09 (0.06-0.13)	14679.09 (10493.41-21907.58)	2.63 (1.88-3.92)	12355.11 (8521.46-19251.83)	4.58 (3.16-7.12)	2323.98 (1521.28-3129.19)	0.82 (0.54-1.11)	12823.15 (9190.30-18535.54)	2.31 (1.65-3.33)	9400.72 (6409.31-14464.82)	3.49 (2.38-5.36)	3422.43 (2274.84-4644.14)	1.21 (0.80-1.65)
Southeast Asia	407.97 (294.97-533.28)	0.23 (0.17-0.30)	272.91 (180.94-382.18)	0.31 (0.21-0.44)	135.05 (97.98-174.26)	0.15 (0.11-0.19)	3691.27 (2666.81-4835.01)	2.06 (1.49-2.70)	2488.02 (1649.02-3493.29)	2.85 (1.89-3.99)	1203.25 (872.02-1549.85)	1.32 (0.96-1.71)	2176.55 (1697.53-2665.90)	1.23 (0.96-1.50)	951.44 (676.24-1286.73)	1.09 (0.78-1.48)	1225.11 (895.67-1540.62)	1.35 (0.99-1.70)
Southern Latin America	30.35 (24.45-37.62)	0.19 (0.16-0.24)	16.92 (12.78-22.16)	0.22 (0.17-0.29)	13.43 (9.80-18.10)	0.17 (0.12-0.23)	276.16 (222.40-342.17)	1.76 (1.42-2.18)	154.57 (116.83-202.47)	2.00 (1.51-2.61)	121.59 (88.86-163.62)	1.53 (1.12-2.07)	139.09 (115.20-166.92)	0.89 (0.74-1.07)	57.44 (44.95-73.36)	0.74 (0.58-0.95)	81.65 (63.13-102.43)	1.03 (0.80-1.30)
Southern Sub Saharan Africa	45.84 (33.62-62.57)	0.20 (0.14-0.27)	32.74 (21.19-48.51)	0.28 (0.18-0.42)	13.10 (9.72-17.39)	0.11 (0.08-0.15)	408.29 (299.37-556.48)	1.75 (1.29-2.39)	294.05 (190.35-434.33)	2.54 (1.64-3.75)	114.24 (84.65-151.47)	0.98 (0.72-1.29)	453.18 (334.95-611.11)	1.95 (1.44-2.62)	270.16 (173.18-416.06)	2.33 (1.50-3.59)	183.01 (135.19-240.90)	1.56 (1.15-2.06)
Tropical Latin America	83.95 (73.65-95.46)	0.17 (0.15-0.19)	47.00 (40.81-53.84)	0.19 (0.16-0.22)	36.96 (31.30-43.26)	0.14 (0.12-0.17)	759.01 (665.56-863.88)	1.51 (1.32-1.72)	427.80 (371.43-490.33)	1.73 (1.50-1.98)	331.21 (280.44-388.18)	1.29 (1.09-1.52)	538.00 (471.00-610.61)	1.07 (0.93-1.22)	214.10 (186.81-242.73)	0.87 (0.76-0.98)	323.90 (276.50-377.34)	1.27 (1.08-1.48)
Western Europe	127.19 (113.49-143.89)	0.18 (0.16-0.20)	48.50 (43.14-54.79)	0.14 (0.12-0.15)	78.69 (67.44-92.27)	0.21 (0.18-0.25)	1168.13 (1042.53-1321.41)	1.61 (1.44-1.83)	445.00 (395.74-502.76)	1.25 (1.11-1.41)	723.13 (619.73-847.71)	1.96 (1.67-2.30)	309.78 (272.58-355.62)	0.43 (0.38-0.49)	105.98 (92.09-123.20)	0.30 (0.26-0.35)	203.80 (176.31-238.63)	0.55 (0.48-0.65)
Western Sub Saharan Africa	77.83 (47.75-113.84)	0.04 (0.03-0.06)	38.03 (24.42-59.05)	0.04 (0.03-0.06)	39.80 (21.63-58.98)	0.04 (0.02-0.06)	681.08 (416.39-1000.91)	0.36 (0.22-0.53)	339.77 (218.65-527.98)	0.37 (0.24-0.57)	341.30 (183.39-507.24)	0.35 (0.19-0.52)	1078.55 (661.61-1539.92)	0.56 (0.35-0.81)	345.30 (224.28-532.46)	0.38 (0.24-0.58)	733.25 (413.68-1062.90)	0.75 (0.42-1.08)

caTC, Thyroid cancer in children and adolescents; ASR, age-standardized rates.

### Trends in incidence, prevalence, and DALYs

**Figure 2:**
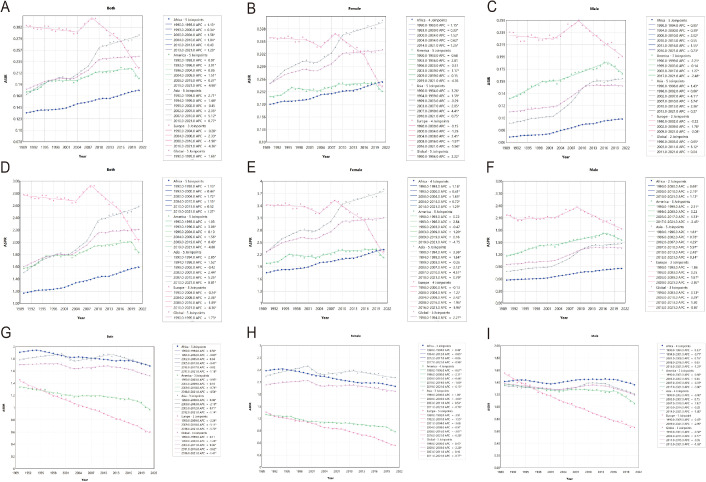
Joinpoint regression analysis of ASIR(ABC), ASPR (DEF) and ASDR(GHI) of caTC at the global and continental (Global-purple-red, Africa-Blue, America-Green, Asia-Gray, Europe-pink) levels from 1990 to 2021. ASIR, age-standardized incidence rate; ASPR, age-standardized prevalence rate; ASDR, age-Standardized Disability-Adjusted Life Years.

Our study carefully illustrated trends of caTC in 204 countries and territories, showing clear geographic variations in incidence, prevalence, and DALYs. Globally, the trend of ASR of incidence showed an increasing pattern, with an AAPC of 1.00. The largest change was observed during the period 2007-2010 (APC, 3.11) (**Supplementary Table S3**). **Figure 1B** graphically represents the estimated annual percentage changes in incidence for various global regions. At the region level, seven regions experienced a notable decrease trend of AAPC, including Western Europe (AAPC, -1.86), Central Europe (AAPC, -1.79), Eastern Europe (AAPC, -0.89), Australasia (AAPC, -0.76), Central Asia (AAPC, -0.75), High-income Asia Pacific (AAPC, -0.70), High-income North America (AAPC, -0.40). On the contrary, the incidence in the remaining regions increased to varying degrees, with South Asia experiencing the highest increase (AAPC, 2.03). Similarly, substantial variations of national-level trends were exhibited among countries and territories, with notable fluctuations observed in different periods. Cabo Verde experienced the greatest increase, with an AAPC of 8.59. Compared to United Kingdom, which experienced the largest decrease, with an AAPC of -3.60 (**Figure 1D**; **Supplementary Tables S3**, **S6**). In terms of continental levels, the ASIR for both males and females was on the rise in Africa, Asia, and America, while it was declining in Europe. Although the ASIR in America was increasing, it began to show a noticeable downward trend at the end of 2019. The ASIR for both males and females was rising fastest in Asia and slowest in America(**Figures 2A-C**; **Supplementary Table S9**).

In the same way, from 1990 to 2021, the trend of TC prevalence among children was found to increase, with an AAPC of 1.05. The greatest changes in ASPR were analyzed during the period 2007-2010 (APC, 3.19). Further analysis by gender revealed that the prevalence rates were increasing for both females (AAPC, 1.64) and males (AAPC, 2.04). Similar to the results for incidence, at the national level, the Cabo Verde (AAPC: 8.69) had experienced the greatest increase in prevalence, while the United Kingdom (AAPC, -3.58) had seen the largest decrease(**Supplementary Tables S4**, **S7**). At the continental level, the ASPR for both males and females was on an upward trend in Africa, Asia, and America, while it was on a downward trend in Europe. The increase in ASPR was most pronounced in Asia for both females and males, and least pronounced in America (**Figures 2D-F**; **Supplementary Table S10**).

The global trend of DALYs was on a declining path (AAPC, -0.37), with similar trends observed for both females (AAPC, -0.28) and males (AAPC, -0.44). The most pronounced decrease in global DALYs occurred between 2016 and 2021 (APC, -1.41). However, significant regional disparities still persisted. Only three regions exhibited an upward trend, including South Asia (AAPC, 0.12), Oceania (AAPC, 0.35), Southern Sub-Saharan Africa (AAPC, 0.65). While the remaining 18 regions showed a downward trend, with the Central Europe experiencing the largest decrease (AAPC, -4.11). In terms of territories and countries, the United Kingdom (AAPC, -4.91) experienced the largest decrease in DALYs, whereas Cabo Verde (AAPC, 6.66) saw the greatest increase (**Supplementary Tables S5**, **S8**). At the continental level, the ASDR for both males and females was on a downward trend in Africa, Asia, America, and Europe, with a particularly pronounced decrease in Europe. For females, the ASDR was declining most rapidly in Europe and most slowly in Asia. For males, the ASDR was also declining fastest in Europe but most slowly in Africa(**Figures 2H-J**; **Supplementary Table S11**).

### Cross-country inequality analysis

**Figure 3:**
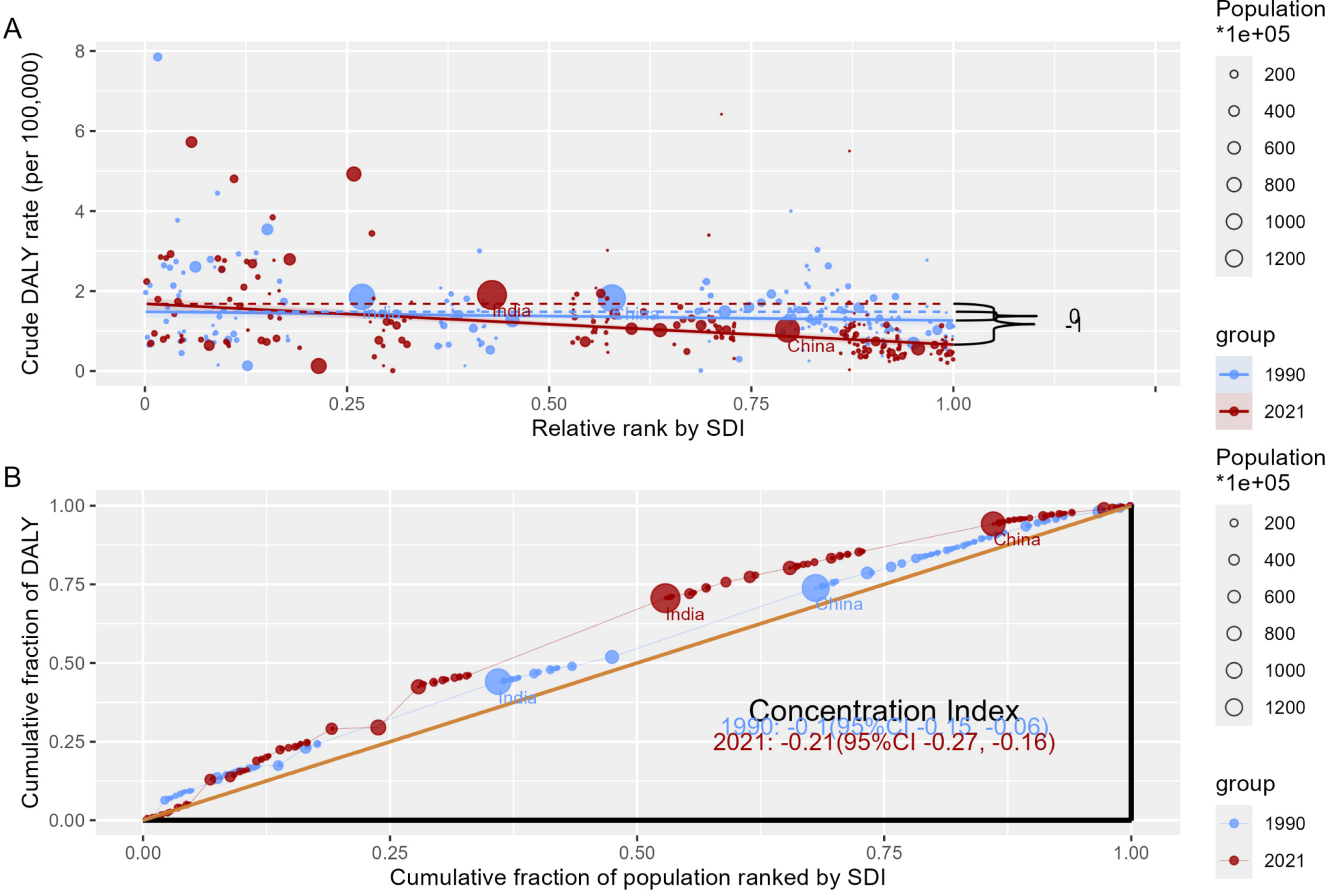
Health inequality regression curves **(A)** and concentration curves **(B)** for the DALYs of caTC from 1990 to 2021 across the world.

Significant disparities in the burden of caTC, both in absolute and relative terms, were evident across countries with varying SDI. A disproportionately higher number of DALYs was concentrated in countries with lower SDI. The slope index of inequality emphasized a widening gap in DALYs rates between countries with the highest and lowest SDI, with values gradually increasing from -0.27 (95% CI: -0.54, -0.01) in 1990 to -1.04 (95% CI: -1.27, -0.80) in 2021 (**Figure 3A**). Furthermore, the concentration index revealed an initial value of -0.10 (95% CI: -0.15, -0.06) in 1990, which subsequently decreased further to -0.21 (95% CI: -0.27, -0.16) in 2021, signifying changes in the distribution of the TC burden across different SDI levels (**Figure 3B**).

### Decomposition and frontier analysis of incidence, prevalence, and DALY rates

**Figure 4:**
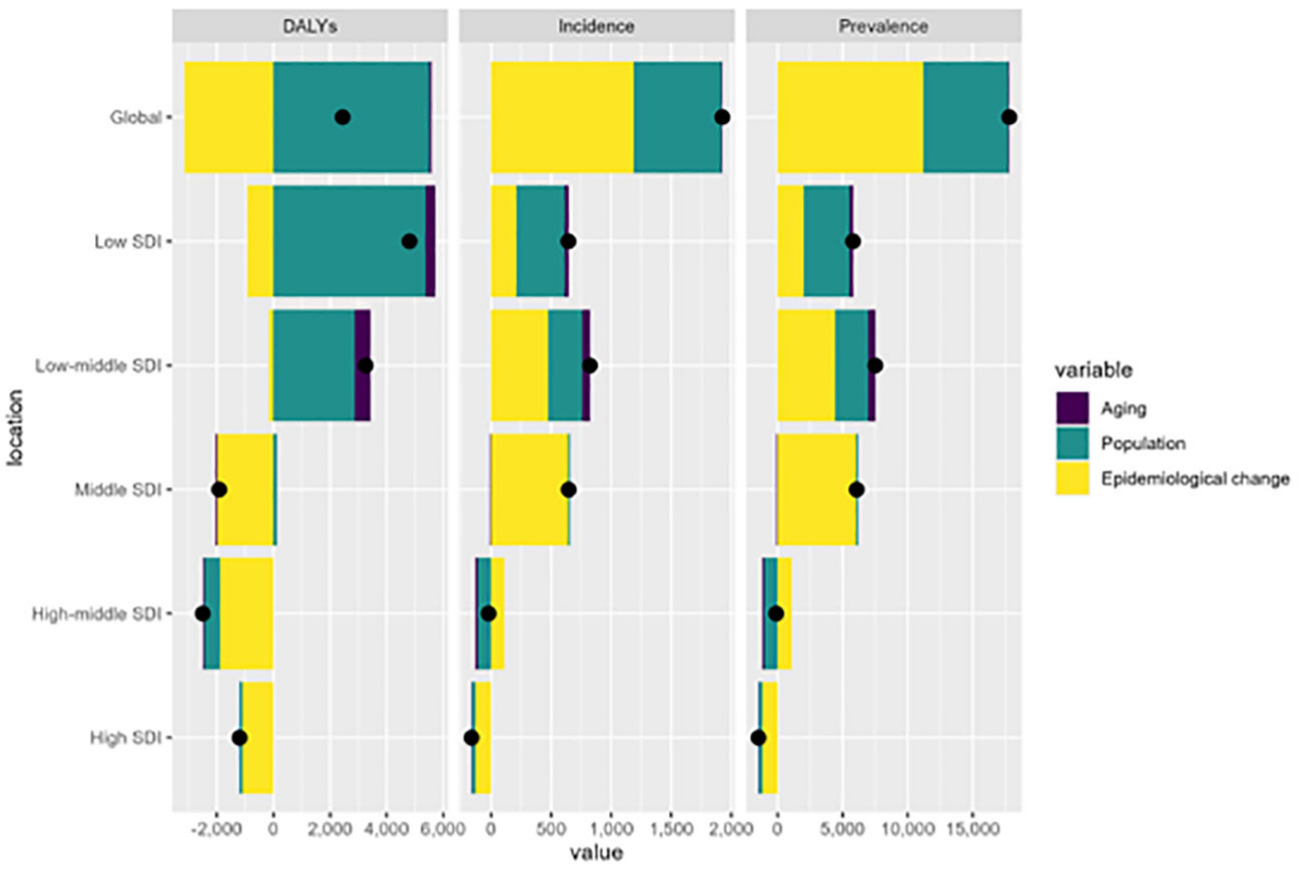
Decomposition analysis of incidence, prevalence, and DALYs. A black dot represents the overall change caused by the three components (population growth, aging, and epidemiological alteration). For each component, an increase in the incidence, prevalence, and DALYs of caTC associated with that component is indicated by a positive value. A decrease in the incidence, prevalence, and DALYs of caTC associated with that component is indicated by a negative value. caTC, thyroid carcinoma among children and adolescents; ASR, age-standardized rate; DALYs, disability adjusted life-years; SDI, sociodemographic index.

Decomposition analysis indicated that over the last 30 years, there has been a notable surge in global incidence and prevalence, with the most substantial rise seen in regions within the low-middle SDI quintile. Epidemiological change was the primary factor, accounting for 57.50% and 58.79% of the changes in incidence and prevalence, respectively. The global DALYs have also risen, albeit not as sharply as the incidence and prevalence. The increase was most pronounced in regions with low SDI. Population growth and aging were the main factors, contributing 88.11% and 17.09% to the change in DALY, respectively (**Figure 4**; **Supplementary Table S12**).

**Figure 5:**
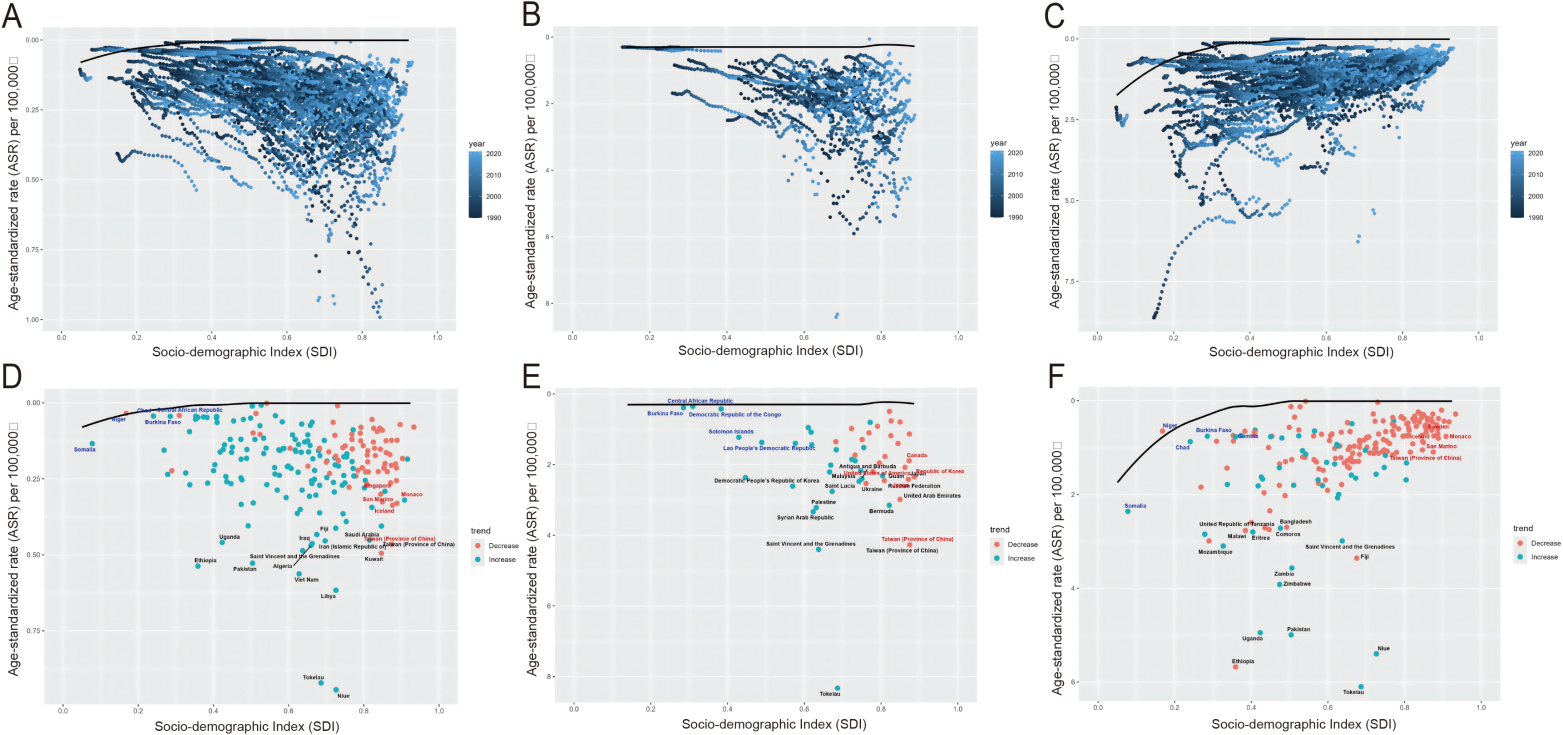
Frontier analysis of SDI and the TC burden in children and adolescents in 2021. **(A, D)** Frontier analysis for ASIR. **(B, E)** Frontier analysis for ASPR. **(C, F)** Frontier analysis for age-standardized rate of DALYs. The frontier is delimited with solid black, and countries and territories are represented by dots. The top 15 countries with the largest significant differences are marked in black (the largest ASR of children and adolescents with an TC gap from the frontier). Countries at the frontier with low SDI (<0.05) and low significant differences are represented in blue (e.g., Somalia, Niger, Nepal, Bangladesh, and the Gambia). While countries and regions with high SDI (>0.85) and relatively high significant differences in development levels are represented in red (e.g., Somalia, Niger, Nepal, Bangladesh, and the Gambia). The increase in age-standardized rates between 1990 and 2021 is indicated by red dots. The decrease in age-standardized rates between 1990 and 2021 is indicated by blue dots. caTC, thyroid carcinoma among children and adolescents; ASIR, age-standardized incidence rate; ASPR, age-standardized prevalence rate; DALYs, disability adjusted life-years; SDI, sociodemographic index. (For interpretation of the references to colour in this Figure legend, the reader is referred to the web version of this article.

**Figures 5A-C** depicts the unfulfilled health improvement potential across regions or nations with varying degrees of development from 1990 to 2021. Meanwhile, **Figures 5D-F** and **Supplementary Table S13** provide insights into the incidence, prevalence, and DALYs burdens in 2021 for countries or regions at different stages of sociodemographic development, as well as the effective disparities between them. As sociodemographic development advances, the effective disparities have generally widened to some degree, indicating that countries or regions with lower SDI possess limited potential for burden reduction.

## Discussion

We conducted a comprehensive analysis of global data from GBD 2021 to examine the current status and temporal patterns in the incidence, prevalence, and disease burden of thyroid cancer among children and adolescents. A cross-national comparative assessment was also performed to evaluate disparities. This investigation covered global, continental (Africa, America, Asia, and Europe), and national levels across 204 countries and regions. Key strengths include the use of extensive datasets, a long-term observational window, and wide geographical coverage. To adjust for variations in population age structures, age-standardized rates (ASIR, ASPR, ASDR) were applied.

Our analysis revealed that in 2021, South Asia accounted for the highest share of global incidence, prevalence, and DALYs related to caTC, representing approximately one-third of total cases. Regions with lower SDI experienced higher ASDR, and this disparity was progressively widening. Compared to males, females exhibited higher ASRs for incidence, prevalence, and DALYs. Trend analysis revealed that the global ASIR of caTC was on the rise, while ASDR was declining, suggesting a potential younger age onset of caTC. At the continental level, the ASIR was increasing in Asia, Africa, and America, but decreasing in Europe. The ASDR in Africa, Asia, America, and Europe was all showing a downward trend, with Europe exhibiting the most pronounced decline. This divergent trend between incidence and mortality/DALYs underscores the complex interplay between enhanced diagnostic intensity (potentially leading to overdiagnosis of indolent cases) and genuine improvements in survival. Decomposition analysis indicated that changes in incidence and prevalence were primarily driven by epidemiological changes, while changes in DALYs were a consequence of population growth. Frontier analysis reveals even greater disparities between countries and regions, highlighting that sociodemographic development alone does not determine health outcomes, and that healthcare system efficiency plays a critical role.

Previous research had indicated that in studies of the thyroid cancer disease burden across all age brackets, East Asia had the greatest number of incidence cases, while Oceania has the lowest. In terms of DALYs, South Asia tops the list, with Oceania again at the bottom, which was partially consistent with our findings (26). From 1990 to 2021, both the ASIR and ASPR of caTC showed an upward trend globally, with a significant surge between 2004 and 2010, followed by a more gradual increase from 2010 to 2021. Several factors could account for this trend. The widespread adoption of neck ultrasound and ultrasound-guided fine-needle aspiration biopsy led to a notable rise in the ASPR for thyroid cancer from 2004 to 2009 (27). Additionally, radiation exposure among children and adolescents remains a crucial environmental risk factor for thyroid cancer, exacerbated by the rising use of computed tomography (CT) scans (28, 29). Furthermore, shifts in dietary patterns and the rising prevalence of obesity may also play a role in the escalating incidence of thyroid cancer (30, 31). From a clinical standpoint, these trends underscore the importance of judicious diagnostic imaging in younger populations and the need for clearer guidelines on thyroid nodule evaluation in pediatric and adolescent patients to avoid overdiagnosis. While the ASIR had generally increased across most regions, notable decreases were observed in certain areas, particularly in Central Europe, Western Europe, and the High-income Asia Pacific region. Nevertheless, during this same period, the ASDR attributed to caTC experienced a decline. This reduction could be traced back to advancements in the diagnosis and treatment of thyroid cancer in numerous countries, ultimately enhancing the overall quality of care (32, 33). The gradual decline in mortality rates could be attributed to the enrichment of cancer treatment approaches and techniques, thanks to the emergence of a diverse array of new diagnostic methods and therapeutic drugs (34).

At the continental level, from 1990 to 2021, the results indicated that there had been a notable upward trend in ASIR and ASPR of caTC in Africa and Asia, while a distinct downward trend was observed in Europe. Although America exhibited a slight overall upward trend, it had shown a significant downward trend since 2019. Around 2015, there were updates to the thyroid cancer screening guidelines in Europe and America, recommending against biopsy for certain nodules (35–37). This recommendation likely contributed to a reduction in new confirmed cases. Hence, overdiagnosis could be a factor contributing to the observed rise in ASIR of TC in Africa and Asia (35, 38). It was worth noting that, with regard to ASDR, our study indicates a decreasing trend across all regions, with Europe exhibiting the most significant reduction. However, a study reported in 2024 on the global disease burden of thyroid cancer across all age groups concluded that the ASDR in Europe had notably decreased, whereas ASDR in Asia, Africa, and the Americas showed an upward trend. This conclusion differed from ours. This discrepancy may suggest that, for thyroid cancer, the disease burden among adults and older individuals was further increasing in Asia, Africa, and America, while it was gradually decreasing among children and adolescents. This divergence highlights the need for age-specific clinical guidelines and public health strategies. In younger cohorts, de-escalation of treatment for low-risk tumors and active surveillance may be increasingly relevant, whereas in older adults, more aggressive case-finding and treatment may still be warranted. Notably, there is a growing consensus that many thyroid cancer cases, particularly papillary thyroid cancer, are low-risk and do not necessitate aggressive or immediate treatment. This understanding has prompted changes in screening, diagnosis, and treatment approaches. Indeed, recent years have witnessed a marked slowdown in the rate of increase for thyroid cancer incidence, reflecting a more cautious and measured approach to managing this disease (37).

The relationship between SDI and caTC burden is complex. In regions with a high SDI, the elevated incidence of caTC could be attributed to their advanced economic status, which facilitates access to superior healthcare services and treatments. This accessibility enhances the availability of diagnostic and therapeutic tools, subsequently leading to an increased detection of cases. Conversely, in low-SDI regions, the thyroid cancer burden is heightened due to, potentially, constraints in healthcare resources, including the lack of advanced medical services and precise laboratory tests (39). This disparity calls for tailored global health initiatives: in high-SDI settings, the focus should be on refining diagnostic criteria to reduce overdiagnosis, whereas in low-SDI settings, the priority is strengthening foundational healthcare capacity to ensure timely diagnosis and access to essential treatments like surgery and radioactive iodine.

We observed that incidence, prevalence, and DALYs of caTC were roughly two times higher in females than males. Potential explanations for this sex disparity include the influence of female reproductive hormones (40). Estrogen has been shown to stimulate thyroid growth. Moreover, differences in healthcare utilization and reproductive health visits may lead to earlier and more frequent diagnosis in females. Clinically, these findings support sex-specific risk assessment and suggest that young female patients may benefit from earlier screening in high-risk settings, though caution is needed to avoid unnecessary procedures. We conducted a frontier analysis at the national level, utilizing data pertaining to ASIR, ASPR, ASDR, and SDI. Our findings highlight considerable opportunities to enhance the management of thyroid cancer’s impact on children and adolescents. Notably, low-SDI countries, including Somalia, Niger, Burkina Faso, and Chad, have succeeded in managing this disease burden despite limited resources. These nations serve as notable examples, showcasing efficient tactics to improve health outcomes even with limited resources. Conversely, high-SDI countries such as Iceland, Monaco, and San Marino have lagged behind in addressing the burden of caTC relative to their developmental status. Variables such as geographic locale and dietary patterns might play a role in this discrepancy. Thus, this highlights the pressing need for enhanced policy formulation and implementation in these regions to optimize and reform healthcare systems.

The incidence, prevalence, and DALYs of caTC exhibited substantial variations across 204 countries, mirroring disparities in prevention, management, and treatment. The results of this study underscored these differences and offered epidemiological insights that can inform the development of public health policies. However, there are several limitations to this study. A crucial limitation of the GBD database is its dependence on health information reporting, as inadequate healthcare systems in some less developed countries may result in potential misdiagnosis and underreporting of cases. Furthermore, it is important to note that for rare pediatric conditions like caTC, particularly in very small populations, the estimates may exhibit instability due to limited raw data. The GBD methodology relies heavily on model-based imputation and smoothing techniques to address data gaps, which introduces a degree of uncertainty. Consequently, the presented age-standardized rates should be interpreted as refined model-derived estimates rather than direct observations from comprehensive registration systems (41). Additionally, the GBD database suffers from inadequate data on thyroid cancer, including the lack of pathological subtypes, tumor staging information, and data on various risk factors, thereby limiting our capacity for further investigation. Last but not least, the variation in diagnostic intensity may influence the observed incidence rates of pediatric thyroid cancer, potentially leading to overdiagnosis of indolent cases. However, the GBD study dataset lacks direct data on diagnostic practices, such as the utilization rates of imaging examinations, making it difficult to conduct more nuanced adjustments. Given this, future research should incorporate data at the individual or registry level and include direct measurement indicators of diagnostic activities to more precisely clarify these influences.

The findings of this study carry significant implications for pediatric endocrinologists, oncologists, and public health policymakers. Firstly, the rising incidence in many regions, potentially fueled by overdiagnosis, calls for a critical re-evaluation of local diagnostic practices. Clinicians should be advocates for the adherence to evidence-based guidelines (e.g. ATA guidelines) that recommend against routine biopsy of small, low-suspicion nodules in children, thereby mitigating the harms of overtreatment. Secondly, the profound inequality in DALYs, concentrated in low-SDI regions, underscores a global disparity in access to essential care, including surgery, radioactive iodine therapy, and thyroid hormone replacement. The global oncology community must support initiatives aimed at bridging this gap by improving infrastructure and training in resource-limited settings.

## Conclusion

Our research provided a thorough examination of the global burden of caTC from 1990 to 2021. Our results indicated a global rise in both the incidence of thyroid cancer cases and the ASIR. The increase has exacerbated the pressure on healthcare systems across the globe, notably in nations with a high SDI and among the female population. Fortunately, the ASDR of caTC had declined, possibly due to advancements in treatment. Regions with lower SDI often bear a heavier burden of caTC, potentially due to poor environmental conditions, unhealthy lifestyles, low health awareness, and ineffective disease control strategies. It is imperative to develop and execute focused strategies and efficacious interventions to diminish the impact of thyroid cancer and halt its escalating trajectory.

## Data Availability

The original contributions presented in the study are included in the article/**Supplementary Material**. Further inquiries can be directed to the corresponding author.

## Supplementary material

Supplementary Table 1 Classification of countries according to GBD region, SDI quintile.

Supplementary Table 2 Female-to-male ratios of the ASIR, ASPR, and ASDR of caTC in 2021.

Supplementary Table 3 Averaged annual percentage changes (AAPCs) of incidence among 204 countries and territories.

Supplementary Table 4 Averaged annual percentage changes (AAPCs) of prevalence among 204 countries and territories.

Supplementary Table 5 Averaged annual percentage changes (AAPCs) of DALYs among 204 countries and territories.

Supplementary Table 6 Joinpoint regression analysis of ASIR of caTC at the national levels from 1990 to 2021.

Supplementary Table 7 Joinpoint regression analysis of ASPR of caTC at the national levels from 1990 to 2021.

Supplementary Table 8 Joinpoint regression analysis of ASDR of caTC at the national levels from 1990 to 2021.

Supplementary Table 9 Joinpoint regression analysis of ASIR of caTC at the global and continental levels from 1990 to 2021.

Supplementary Table 10 Joinpoint regression analysis of ASPR of caTC at the global and continental levels from 1990 to 2021.

Supplementary Table 11 Joinpoint regression analysis of ASDR of caTC at the global and continental levels from 1990 to 2021.

Supplementary Table 12 Incidence, Prevalence, and DALYs of caTC with decomposition analysis, categorized by global and SDI regions.

Supplementary Table 13 Age-standardized Incidence, Prevalence, and DALYs rates of caTC with frontier analysis across all countries and territories.
